# Development and Characterization of Transgenic Sugarcane with Insect Resistance and Herbicide Tolerance

**DOI:** 10.3389/fpls.2017.01535

**Published:** 2017-09-28

**Authors:** Wen Zhi Wang, Ben Peng Yang, Xiao Yan Feng, Zheng Ying Cao, Cui Lian Feng, Jun Gang Wang, Guo Ru Xiong, Lin Bo Shen, Jun Zeng, Ting Ting Zhao, Shu Zhen Zhang

**Affiliations:** Institute of Tropical Bioscience and Biotechnology, Chinese Academy of Tropical Agricultural Sciences, Haikou, China

**Keywords:** sugarcane molecular breeding, EPSPS gene, Cry1Ab gene, PMI/mannose selection, *Agrobacterium*-mediated transformation

## Abstract

Genetically modified crops which had been commercial applied extensively majorly are the insect resistance and herbicide tolerance events. In this study, the Bt insecticidal gene Cry1Ab, the glyphosate-tolerant gene EPSPS, and the selection marker gene PMI were combined into a single transferred DNA fragment and introduced into sugarcane by *Agrobacterium*-mediated transformation. Thirty-three resistant plantlets were obtained after selection using a PMI/mannose selection system. Thirty of these resistant plantlets were PCR positive for the three target genes. Southern blot assay revealed that the copy number of the integrated fragment in the transformed plantlets varied from 1 to 7. ELISA analysis showed that 23 of the 33 resistant plantlets expressed Cry1Ab and EPSPS protein. Five single-copy and ELISA-positive transgenic lines were tested under laboratory and field conditions to determine their resistance to insects and herbicides, and also evaluated their agronomic characteristics and industrial traits. Results showed that larvae fed with fodder mixture containing stem tissues from single-copy transgenic lines were weak and small, moreover, pupation and eclosion were delayed significantly during voluntary feeding bioassays. None of transgenic sugarcane was destroyed by cane borer while more than 30% of wild type sugarcane was destroyed by cane borer. For herbicide resistance, the transgenic plantlets grew healthy even when treated with up to 0.5% roundup while wild type plantlets would die off when treated with 0.1% roundup. Thus demonstrate that these transgenic lines showed strong insect resistance and glyphosate tolerance under both laboratory and field conditions. But in the field most of the transgenic plants were shorter and more slender than non-transformed control plants. So they presented poor agronomic characteristics and industrial traits than non-transformed control plants. Thus, a considerable number of embryogenic calli should be infected to obtain transgenic lines with potential for commercial use.

## Introduction

Herbicide tolerance and insect resistance are important traits considered in genetic improvement of various crops (James, 2013). Herbicide-tolerant transgenic crops represent the majority of genetically modified plants cultivated worldwide. In 2010, about 83% of the 148 million ha land dedicated for genetically modified crops was planted with herbicide-tolerant species (James, 2010; Bonny, 2011). Introduction of insect-resistant genes, such as Bt genes, is an effective and economic strategy used to improve the resistance of various crops to insects (Dutton et al., 2003; Torres and Ruberson, 2006; Valderrama et al., 2007; Gatehouse, 2008; Srikanth et al., 2011; Karthikeyan et al., 2012). Currently, commercially grown GM crops contain genes related to insect resistance and herbicide tolerance, which are valuable traits in production of major crops, such as corn and cotton. Transgenic plants stacked with multiple genes are mostly obtained by cross-hybridization among different transgenic plants (Cao et al., 2002; Datta et al., 2002; Zhao et al., 2003) or re-transformation with different genes (Jobling et al., 2002; Rosati et al., 2003; Singla-Pareek et al., 2003; Qi et al., 2004). However, these methods for combining transgenes present several significant limitations. For example, cross-hybridization is time consuming and labor intensive, transgenic lines segregate again in subsequent generations (Halpin et al., 2001; Halpin, 2005). In this regard, scholars have developed a fast and cost effective method of introducing one single transferred DNA (T-DNA) containing multiple genes, with each gene possessing its own promoter and terminator (Slater et al., 1999; Bohmert et al., 2000, 2002). This method can be used to introduce different genes into a plant by inserting them at the same site of the genome through one transformation cycle. Nevertheless, linking multiple genes into one T-DNA exhibits limitations in terms of T-DNA size and uneven gene expression among different genes (Jones et al., 1987; Peach and Velten, 1991). Thus, this method must be further improved (Poirier et al., 2000; Ye et al., 2000; Goderis et al., 2002; Thomson et al., 2002).

Sugarcane (*Saccharum officinarum* L.) is an important sugar crop that is widely cultivated in the tropical and subtropical regions. It provides about 80% of the world sugar (Raza et al., 2010) and more than 92% of sugar in China (Guo et al., 2013). Equally important is the sugarcane borer, *Diatraea saccharalis* (F.), which is one of the most important lepidopteran pests attacking sugarcane plants and causing more than 10% loss in sugarcane yield worldwide (Ricaud et al., 1989). At the other hand, for sugarcane is mostly planted at rainfed cropland so it usually suffered badly by weed infestation and induce yield lost too. There are a lot of reports that introduced Bt gene to sugarcane cultivar and successfully obtained insect resistant sugarcane transgenic lines (Gao et al., 2016). Also, some reports introduced PAT/bar gene to sugarcane cultivar and obtained transgenic lines which resistant to Basta (glufosinate-ammonium) (Falco et al., 2000). But by now, there is no report that introduced Bt gene and EPSPS gene together to sugarcane cultivar and try for commercial used.

In this study, our goal was to check the possibility of transformed Cry1Ab and EPSPS gene together to sugarcane commercial cultivar, and to obtain transgenic lines that resistant to cane borer and herbicide. So a plant expression vector containing a Cry1Ab gene, an EPSPS gene and a selection marker gene PMI was adopted and transformed to a sugarcane commercial cultivar by agro-bacterium media transformation method. Embryogenic calli of this sugarcane cultivar were used as explant material and infected by agro-bacterium containing the plant expression vector. Resistant plants were obtained after selection by different kinds of medium containing mannose. Molecular characterization of these resistant plants was conducted by PCR, Southern blot and analysis. And then single copy and ELISA positive transgenic lines were chose for lab and field testing to determine the cane borer and herbicide resistant ability. Agronomic and industrial traits in field of these single copy transgenic lines were evaluated too. It is anticipated that the findings of this study will allow molecular breeding of sugarcanes that are resistant to cane borers and herbicide. But poor agronomic traits of these transgenic lines induce the difficult of commercial use.

## Materials and Methods

### Binary Vectors and Agrobacterium Strain

**FIGURE 1:**
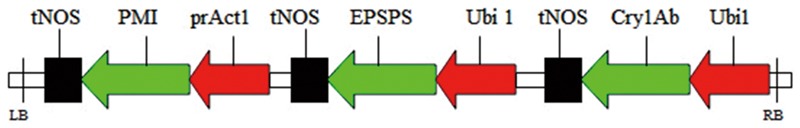
Schematic of the T-DNA vector used for sugarcane transformation. PMI gene promoted by the prAct-1 promoter and the Cry1Ab gene and the EPSPS gene were promoted by the prUbi-1promoter. prUbi-1, maize Ubi-1 promoter; prAct-1, rice Act-1promoter; tNOS, nopaline synthase terminator.

The expression vector harboring the Cry1Ab gene, EPSPS gene, and the selection marker gene PMI was constructed in our previous research and stored in our laboratory. The Cry1Ab gene and the EPSPS gene were promoted by the Ubi-1 promoter and terminated by the nopaline synthase terminator. The selection marker gene was promoted by the Act-1 promoter (**Figure 1**). The expression vector was mobilized into *Agrobacterium* EHA105 through freeze thawing with liquid nitrogen. The *Agrobacterium* strain was cultured on YEP (yeast extract, 10 g/L; peptone, 10 g/L; NaCl, 5 g/L) medium containing appropriate antibiotics (spectinomycin, 100 mg/L; streptomycin, 50 mg/L; and rifampicin, 10 mg/L).

### Explant Material and Callus Induction

The tops of sugarcane tillers containing immature leaf whorl were excised from the plant of variety ROC22 and used as source material for embryogenic callus induction. Transverse sections of immature sugarcane leaf whorls were prepared using the method described by Bower and Birch (1992). Transverse sections of approximately 1 mm thickness were cut immediately above the meristem and placed on callus induction medium [Murashige and Skoog (MS) + 30 g/L sucrose + 2 mg/L 2,4-D and 8 mg/L agar] (Murashige and Skoog, 1962). Callus cultures were maintained at 28°C in the dark and supplied with fresh medium every 2 weeks. After approximately 45 days of induction, faint yellow and hard calli were selected and fragmented prior to transformation.

### Transformation and Selection

Calli for transformation were collected and weighed prior to infection and placed into an Erlenmeyer flask. The *Agrobacterium* cells cultured in YEP medium were collection with centrifugal machine and re-suspended by infection medium (1/5 strength MS medium + 30 g/L sucrose + 30 g/L glucose + 100 mM acetosyringone) and diluted to the concentration about 0.5–1.0 of OD_600_. The flask contained calli was added with liquid infection medium with *Agrobacterium* harboring the expression vector and shaken gently for about 30 min in the dark. The *Agrobacterium* suspension was pumped out. The calli were transferred to a Petri dish, blotted dry to remove excess *Agrobacterium* suspension by using filter paper, and air dried for about 30 min on the cleaning bench. The calli were transferred to a new Petri dish and incubated in the dark at 22°C for 3 days (Dong et al., 2014). The infected calli were transferred to resting medium (MS + 1.0 mg/L 2,4-D + 30 g/L sucrose + 8 g/L agar + 300 mg/L timentin) and cultured in the dark at 28°C for 7 days. All infected calli were subsequently transferred to selection medium (MS + 2.0 mg/L 2,4-D + 8 g/L agar + 300 mg/L timentin + 5 g sucrose + 8 g mannose) and incubated in the dark at 28°C for 35 days. The resistant calli on the dark selection medium were transferred to regeneration medium (MS + 1.0 mg/L 6-BA + 8 g/L agar + 300 mg/L timentin + 5 g sucrose + 8 g mannose) and cultivated in an illuminated incubator for 14 days. After regeneration, green buds were transferred to elongation medium (MS + 8 g/L agar + 300 mg/L timentin + 5 g sucrose + 8 g mannose) and cultivated in an illuminated incubator for 20 days. A single shoot from each of the buds was transferred to fresh elongation medium and cultured for 20 days. Resistant shoots were sampled for molecular analysis and transferred to soil.

### PCR Assay of Resistant Shoots

Total genomic DNA was extracted from the leaves of the resistant shoots by using CTAB method. The vector plasmid was used as positive control, and total genomic DNA extracted from wild-type plants was used as negative control. The concentration of all DNA samples was measured using UV absorption method. DNA purity was analyzed using A260/A280 ratio. Based on the sequences of the Cry1Ab, EPSPS, and PMI genes, three pairs of primers were designed by Primer Premier version 5 for PCR assay (**Table 1**). Each 25 μL of the reaction volume contained 2.5 μL of PCR buffer (10×), 2.0 μL of dNTP mix, 1.0 μL of (100 ng/L) template DNA, 1.0 μL of each forward and reverse primer, 0.2 U Taq DNA polymerase (Takara, Dalian, China), and 17.3 μL of double-distilled H_2_O (ddH_2_O). The PCR reaction was conducted as follows: initial polymerase activation at 98°C for 5 min; 35 cycles of 95°C for 30 s, 58°C for 30 s, and 72°C for 30 s; and final extension at 72°C for 10 min. The PCR products were detected on 1.0% (w/v) agarose gel. PCR analysis of all samples was repeated a least three times to avoid appearance of false positive. Final results were then recorded in the table.

**Table 1:** Primer sequences for PCR analysis.

Gene	Size of PCR production	Primers	
PMI	513bp	Forward	ACTACAAGGACCCCAACCAC
		Reverse	TGGCCTCGAACTTCACGTTG
EPSPS	643bp	Forward	CGCGATCACACCGAGAAGAT
		Reverse	CCCATCACGAGGAACGACAT
Cry1Ab	359bp	Forward	GTTCACCTTCCCCCTGTACG
		Reverse	TAGGTGCACGGATGATGCTC

*All primers for PCR analysis were designed with Primer Premier version 5.0 with the amplified 513-bp DNA fragment for the PMI gene, 643-bp DNA fragment for the EPSPS gene, and 359-bp DNA fragment for the Cry1Ab gene.*

### Southern Blot Analysis

Southern blot analysis was conducted to determine the integration and copy number of the integrated fragments. Approximately 10 μg of total genomic DNA extracted from the transformed sugarcane plants was digested with EcoRI restriction enzyme, separated by electrophoresis agarose gel, and transferred onto Hybond N+ membrane. A 513-bp digoxin-labeled DNA fragment corresponding to the Cry1Ab gene was used as probe for hybridization according to the instruction manual (DIG High Prime DNA Labeling and Detection Starter Kit I, Roche).

### Detection of Cry1Ab and EPSPS Protein by ELISA

All resistant plants were sampled and tested by ELISA. The sample from non-transgenic sugarcane stem was designated as negative control. Briefly, 0.1 g of stem tissues obtained from all samples were cut into small pieces and pushed to the bottom of a 1.5 mL Eppendorf tube. Pestles were inserted into each tube, and the stem tissue samples were ground by rotating the pestles against the sides of the tube with twisting motions. This process was continued until the stem tissue samples were well ground. The samples were then added with 1 mL of 1× phosphate-buffered saline with Tween 20 (PBST) buffer. The grinding step was repeated to mix the tissue thoroughly with 1× PBST extraction buffer. All tubes with the sample mixture were centrifuged for 1 min, and the liquid supernatant was used for ELISA. ELISA testing was conducted using the ELISA Kit (Bt-Cry1Ab/1Ac ELISA Kit, Agdia, United States; CP4-EPSPS ELISA Kit, Agdia, United States) according to the manufacturer’s instructions. The development of blue color in the ELISA mixture indicates positive protein expression. ELISA analysis for all samples was repeated at least three times to avoid appearance of false positive. Final results were then recorded in the table.

### Toxicity Testing of Single-Copy Transgenic Lines

*Helicoverpa armigera* larvae were used to test the toxicity of Cry1Ab protein expression in single-copy transgenic sugarcane stems through volunteer feeding bioassay. All third-instar larvae were starved for 12 h before feeding bioassay was initiated. Briefly, 4 g of fresh sugarcane stem tissue from single-copy transgenic lines was ground into powder with liquid nitrogen and mixed with 40 mL of larva fodder (8 g of larva fodder powder + 40 mL of ddH_2_O + 0.35 g of agar). The larva fodder mixture was equally distributed into 12-well cell culture plate (Cell Culture Plate, 12-well, Eppendorf, Germany). Twelve larvae with similar body size and weight were obtained from each well. The culture plates were covered and placed in an incubator (28 ± 2°C, 70% RH) in the dark. All bioassays were conducted for 1 month. The body weight of each larva was recorded from the first to the eighth day. Average weight and the standard deviation of 12 larvae fed by different larva fodder were analyzed by Excel version 2007. Development and pupation of larvae were also observed for about 1 month.

### Glyphosate Tolerance Testing of Single-Copy Transgenic Lines

The propagated clones of each single-copy transgenic lines were grown in flowerpots and cultured for approximately 2 months until reaching a height of about 40 cm. Subsequently, 25 mL of different dilutions (from 0.1 to 0.5%) of Roundup (Monsanto, St. Louis, MO, United States) were sprayed to each plantlet of the clonal propagation of each transgenic line. Each treatment had three replications. After 10 days, the plantlets were observed, and pictures were taken.

### Evaluation of Agronomic and Industrial Traits in Single-Copy Transgenic Lines

In isolated field trials, single-copy transgenic lines and non-transgenic controls were evaluated from 2014 to 2015 by using a randomized complete block design with 10 replications. Each block possessed one row of 10 m length, with 1.2 m space between rows, covering an area of 12 m^2^. During harvest in January 2015, yield estimates were obtained from the stems of each row. Height, stem diameter, and brix were measured in 10 consecutive principal stalks from 10 plants. The number of millable stalks in each row was also counted. Theoretical cane yield per hectare was calculated based on the area, number, and weight of stalks using equations:

(1) $$\text{Weight}\;\text{of}\;\text{single}\;\text{stalk}\;(\text{kg})\;\text{=}\;\text{1/4}\;\times \;\pi \;\times \;{\left(\text{diameter}\right)}^{\text{2}}\;\times \;\text{Height}\;\times \;\text{1}.\text{0/1000}$$

(2) Number of stalks per hectare = 10000 × Stalk number per row / 12

(3) Theoretical cane yield (t/ha) = Weight of single stalk × Number of stalks per hectare /1000.

Theoretical sucrose yield was calculated based on the average sucrose content and cane yield using equations:

(4) Sucrose content(%)=brix×1.0825−7.703;

and

(5) Sucrose yield (t/ha) = Cane yield (t/ha) × Sucrose content (%)

In the field trials, the percentage of stalks damaged by the sugarcane borer and agronomic traits were recorded. Roundup tolerance in the field was checked by determining the spray concentration of roundup (0.2%).

## Results

### Plant Material and Callus Induction

ROC22 represents the largest acreage of sugarcane cultivars in China; Scholars have focused on improving the resistance of this cultivar to herbicides and insect borers.

**FIGURE 2:**
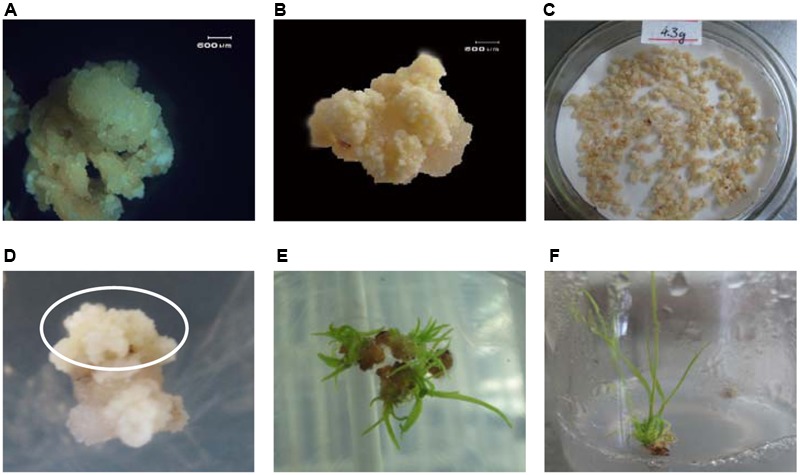
Embryogenic callus induction and transformation. **(A)** Calli induced after 15 days; **(B)**: Embryogenic calli formed after 45 days; **(C)**: Embryogenic calli selected, fragmented, weighed, and air dried prior to transformation; **(D)** After 30 days of dark selection, several healthy resistant calli grew on the selection medium; **(E)** After 14 days of regeneration, healthy cultivation-resistant calli emerged rapidly; **(F)** After 20 days of elongation, cultivation-resistant, dark-green shoots grew quickly.

In this research, 4 tops of sugarcane tillers containing immature leaf whorl were excised from the plantlets of variety ROC22 and used as source material for embryogenic callus induction. Calli grew fast and healthy after 15 days of induction, but the embryogenic calli had not formed (**Figure 2A**). Improved embryogenic calli formed after renewed twice of induction medium as described in material and methods (**Figure 2B**). 4.3 g of faint yellow and hard calli were obtained and fragmented before transformation (**Figure 2C**).

### Transformation and Selection

After 30 min of infection, 3 days of co-cultivation, 7 days of resting cultivation, and approximately 35 days of dark selection, about 5% of infected callus grew out new resistant embryogenic calli (around 5 mm in size) on the selection medium (**Figure 2D**). These resistant embryogenic calli were obtained from the dark selection medium and transferred to the regeneration medium. The somatic embryos soon formed on the resistant embryogenic calli in 7 days. And then monocotyledon formed and finally germinated in follow 7–14 days (**Figure 2E**). Finally, 33 independent resistant shoots (**Figure 2F**) were obtained after elongation selection. The shoots were sampled for PCR analysis.

### PCR and Southern Blot Analyses of Potential Transformed Shoots

A total of 33 resistant shoots were sampled and evaluated for PMI gene, EPSPS gene, and Cry1Ab gene integration by PCR after elongation cultivation. Thirty-two of the shoots were PCR positive for PMI and EPSPS genes, whereas 30 of the shoots were PCR positive for the Cry1Ab gene. A total of 30 transgenic lines were PCR positive for all of the three target genes (**Table 2**). Southern blot analysis was performed for the 33 resistant shoots by using probes specific to the Cry1Ab gene to determine the copy number of the inserted T-DNA. The copy number of the integrated fragment in the transformed lines varied from 1 to 7 (**Table 2**), and transgenic lines T3, T5, T9, T15, and T18 were confirmed to be single-copy inserted events.

**Table 2:** PCR, Southern blot, and ELISA analyses.

Resistant shoots		T1	T2	T3	T4	T5	T6	T7	T8	T9	T10	T11
PCR	PMI	+	+	+	+	+	+	+	+	+	+	+
	EPSPS	+	+	+	+	+	+	+	+	+	+	+
	Cry1Ab	+	+	+	+	+	+	+	+	+	+	+
ELISA	EPSPS	+	+	+	+	+	+	-	+	+	+	+
	Cry1Ab	+	+	+	+	+	+	-	+	+	-	+
Copy No.	Cry1Ab	2	5	1	6	1	3	2	7	1	3	4

**Resistant shoots**		**T12**	**T13**	**T14**	**T15**	**T16**	**T17**	**T18**	**T19**	**T20**	**T21**	**T22**

PCR	PMI	+	+	+	+	+	+	+	+	+	-	+
	EPSPS	+	+	+	+	+	+	+	+	+	-	+
	Cry1Ab	+	+	-	+	+	+	+	+	+	-	+
ELISA	EPSPS	+	-	-	+	-	-	+	+	+	-	+
	Cry1Ab	-	-	-	+	-	-	+	+	+	-	+
Copy No.	Cry1Ab	3	2	0	1	6	2	1	5	4	0	2

**Resistant shoots**		**T23**	**T24**	**T25**	**T26**	**T27**	**T28**	**T29**	**T30**	**T31**	**T32**	**T33**

PCR	PMI	+	+	+	+	+	+	+	+	+	+	+
	EPSPS	+	+	+	+	+	+	+	+	+	+	+
	Cry1Ab	+	+	+	-	+	+	+	+	+	+	+
ELISA	EPSPS	+	+	+	-	+	+	-	+	+	+	+
	Cry1Ab	+	+	+	-	+	+	-	+	+	+	+
Copy NO.	Cry1Ab	4	5	7	0	2	4	3	2	5	6	3

*“+” PCR or ELISA analysis positive; “-” PCR or ELISA analysis negative.*

### ELISA Analysis of EPSPS and Cry1Ab Protein

Double-antibody sandwich ELISA was performed to determine the protein expression levels of EPSPS and Cry1Ab in the stem tissues of all 33 resistant shoots. The development of blue color in the ELISA reaction mixtures indicates positive protein expression. 25 resistant shoots were positive for EPSPS protein expression, and 23 resistant shoots were positive for Cry1Ab protein expression. A total of 23 resistant shoots were positive for both EPSPS and Cry1Ab protein expression (**Table 2**).

### Toxicity Testing of Single-Copy Transgenic Lines

**FIGURE 3:**
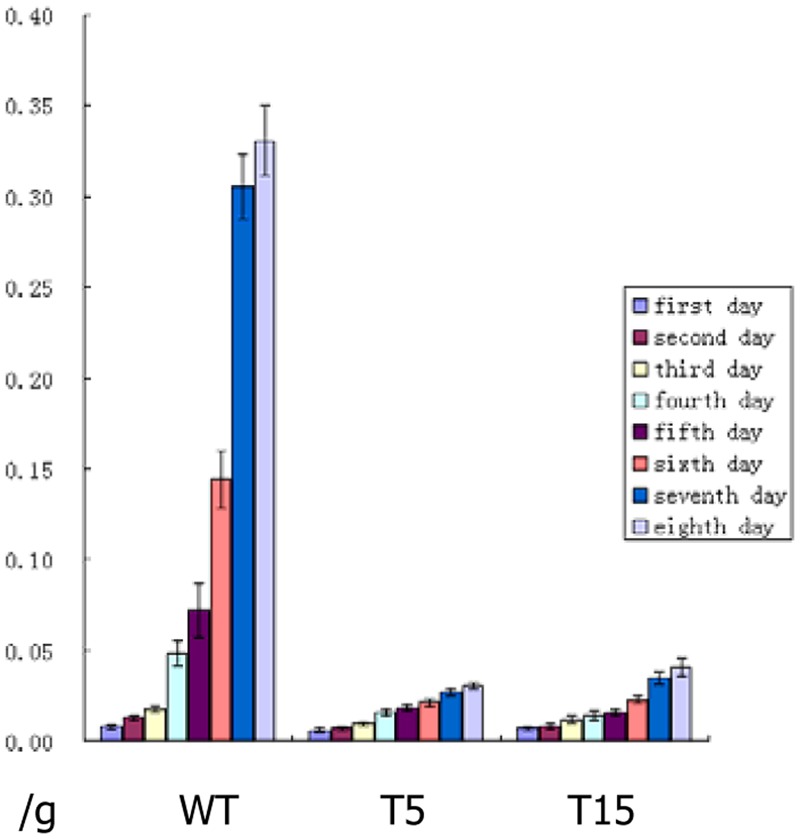
Daily average weight of larvae after feeding bioassay. WT: Daily average weight of 12 larvae fed with larva fodder mixture containing wild-type sugarcane stem tissue, they grew fast from first day to eighth day; T5: Daily average weight of 12 larvae fed with larva fodder mixture containing the T5 transgenic line stem tissue, they grew much slower than wild type from first day to eighth day; T15: Daily average weight of 12 larvae fed with larva fodder mixture containing T15 transgenic line stem tissue, they grew much slower than wild type too from first day to eighth day.

Five single-copy transgenic lines, namely, T3, T5, T9, T15, and T18, were selected to test the toxicity of the Cry1Ab protein expressed in stems. The body weight of each larva was recorded for the first 8 days. Larva pupation and eclosion were observed for 1 month of the bioassay treatment. Larvae fed with fodder mixture containing stem tissues from all single-copy transgenic lines were weak and small; moreover, pupation and eclosion were delayed significantly. T5 and T15 showed the optimal inhibition of larva growth. The average weight of 12 larvae fed with wild-type sugarcane stem tissue increased from 0.0079 to 0.3308 g in the first 8 days. Meanwhile larvae fed with T5 and T15 stem tissues grew from 0.0069 to 0.0306 g and from 0.0072 to 0.0405 g, respectively (**Figure 3**).

**FIGURE 4:**
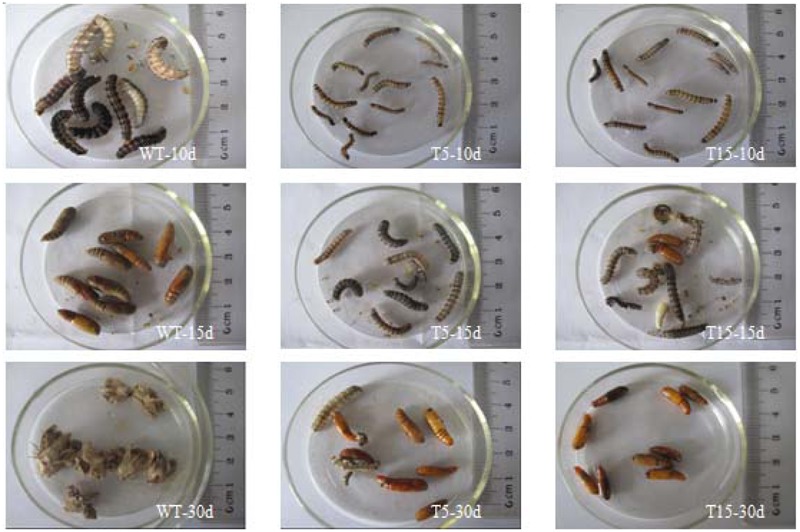
Pupation and eclosion of larvae during feeding bioassay. WT-10d: 10 days after larvae fed by wild type stem tissue; WT-15d: 15 days after larvae fed by wild type stem tissue; WT-30d: 30 days after larvae fed by wild type stem tissue; The left three images shows that larvae feed by wild type stem tissue grow healthy and all larvae underwent eclosion in 30 days. T5-10d: 10 days after larvae fed by T5 stem tissue; T5-15d: 15 days after larvae fed by T5 stem tissue; T5-30d: 30 days after larvae fed by T5 stem tissue. The middle three images shows that larvae feed by T5 stem tissue grew slowly and small, the pupation and eclosion was delayed badly. T15-10d: 10 days after larvae fed by T15 stem tissue; T15-15d: 15 days after larvae fed by T15 stem tissue; T15-30d: 30 days after larvae fed by T15 stem tissue. The right three images shows that larvae feed by T5 stem tissue grew slowly and small, the pupation and eclosion was delayed badly.

At 10 days after feeding bioassay, larvae fed with wild-type stem tissue were strong and fat, whereas larvae fed with T5 and T15 were weak and slender. At 15 days after feeding bioassay, all larvae fed with wild-type stem tissue had pupated, whereas larvae fed with T5 and T15 started to pupate. At 30 days after feeding bioassay, all larvae fed with wild-type stem tissue underwent eclosion, whereas larvae fed with T5 and T15 were in the progress of pupation (**Figure 4**).

### Glyphosate Tolerance Testing of Single-Copy Transgenic Lines

**FIGURE 5:**
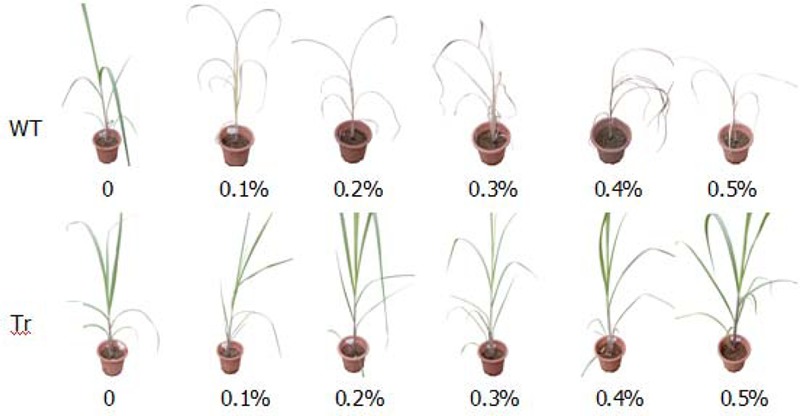
Glyphosate tolerance testing of single-copy transgenic lines. WT: Sugarcane clonal propagation from the wild-type line; Tr: Sugarcane clonal propagation from single-copy transgenic lines. The transgenic clonal grew healthy even when treated with up to 0.5% roundup while wild type clonal would die off when treated with 0.1% roundup.

Ten days after spraying different dilutions of roundup (from 0.1 to 0.5%), all wild-type sugarcane plantlets died even those given with the lowest roundup concentration of 0.1%. The transgenic plantlets grew healthy even when treated with up to 0.5% roundup (**Figure 5**). Hence, the transgenic lines possess high glyphosate tolerance.

### Evaluation of Agronomic and Industrial Traits in Single-Copy Transgenic Lines

The agronomic and industrial traits of the five single-copy transgenic lines and control sugarcane cultivar ROC22 were investigated in mature stage plants. As shown in **Table 3**, univariate statistical analysis indicated that stalk height was low in T9 and T15 but was not significantly different from the control. By contrast, the stalk heights of the three other transgenic lines were significantly lower than that of the control. The stalk diameter of T3 and T18 was smaller but not significantly different from that of the control, whereas the three other transgenic lines presented significantly smaller diameter than the control. In terms of brix, only T18 presented significantly lower values compared with the control, whereas the other lines presented lower but not significantly different values compared with the control. The millable stalk number of each row of T5 and T9 was significantly lower than that of the control, whereas the three other lines showed similar numbers to that of the control. Finally, theoretical cane yield and sucrose yield were calculated based height, diameter, brix, and number of millable stalks in a row. The yield of the five transgenic lines was significantly lower than that of the control.

**Table 3:** Agronomic characteristics, industrial traits, and the stalk borer damage percentage in single-copy transgenic lines and the control lines.

Tr Lines	Height (cm)	Diameter (cm)	Brix (%)	SNR	TCY (t/ha)	TSY (t/ha)	SDR (%)
T3	286.10 ± 16.06c	2.65 ± 0.17ab	20.75 ± 1.09ab	111.90 ± 4.84ab	132.48 ± 5.73d	17.98 ± 0.74d	0
T5	285.20 ± 10.66c	2.49 ± 0.19b	20.5 ± 1.25ab	108.80 ± 3.58b	112.48 ± 3.71e	14.97 ± 0.47e	0
T9	331.9 ± 15.76ab	2.61 ± 0.15b	20.95 ± 1.12a	108.80 ± 3.43b	146.95 ± 4.6c	20.24 ± 0.6c	0
T15	343.90 ± 26.58a	2.66 ± 0.21ab	20.9 ± 0.84ab	113.10 ± 6.42ab	164.08 ± 9.3b	22.51 ± 1.21b	0
T18	278.90 ± 17.15c	2.53 ± 0.294b	20.00 ± 1.08b	113.10 ± 5.24ab	117.87 ± 5.46e	15.09 ± 0.66e	0
CK	356.80 ± 8.87a	2.84 ± 0.18a	20.95 ± 0.72a	114.70 ± 5.38a	197.96 ± 9.28a	27.26 ± 1.21a	31%

*(1) SNR, stalk number per row (10 m); TCY, theoretical cane yield; TSY, theoretical sucrose yield; SDP, stalk damage percentage. (2): Data followed by different letters indicate significant difference at 0.05 level (LSD test).*

**FIGURE 6:**
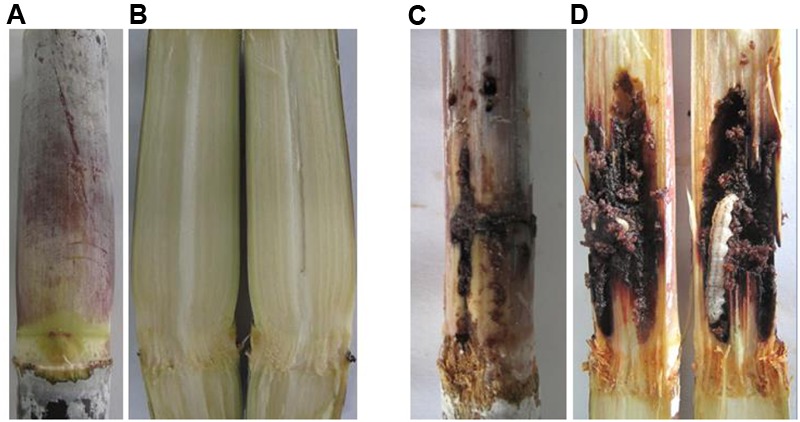
Comparison of cane borer damage between transgenic and control line stalks. **(A)** Stem symptom of transgenic lines (grew healthy and did not destroy by cane borer); **(B)** Stem symptom of transgenic lines (inside stalk); **(C)** Stem symptom of control lines (Destroyed badly by cane borer); **(D)** Stem symptom of control lines (inside stalk grew with cane borer).

**FIGURE 7:**
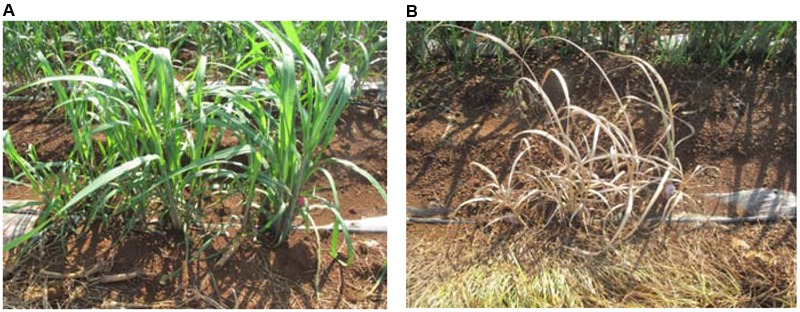
Roundup tolerance testing in field trials. **(A)** Transgenic lines grew healthy 10 days after sprayed by 0.2% Roundup; **(B)** Control lines totally died off 10 days after sprayed by 0.2% Roundup.

In the field trials, none of stalks from the transgenic lines showed cane borer damage, whereas 31% of stalks from the control lines were damaged by cane borer (**Figure 6** and **Table 3**). As observed for the Roundup tolerance testing in the field trials, at 10 days after spraying of 0.2% Roundup, control sugarcane lines died off, whereas the transgenic lines remained dark green and grew healthy (**Figure 7**).

## Discussion

Transgenic crops with more than one trait offer broad agronomic enhancements to meet requirements under complex farming conditions. Herbicide tolerance and insect resistance are important traits considered in genetic improvement of various crops. For sugarcane, yield lost were caused by pests attacking and weed infestation too. So herbicide tolerance and insect resistance are the two important characters in genetic improvement of sugarcane. But by now, there is no report that introduced Bt gene and EPSPS gene together to sugarcane cultivar and try for commercial used.

In this research, we constructed the plant expression vector containing three expression cassettes, Ubi1-Cry1Ab-tNOS, Ubi1-EPSPS-tNOS and prAct1-PMI-tNOS. The two target genes were placed under the control of the constitutively expressed promoter Ubi1 and the selectable marker gene PMI was placed under the control of the promoter Act1. Maize Ubi-1 promoter is a constitutive and strong promoter and had been commonly used in previous sugarcane genetic improvement researches (Falco and Silva, 2003; Zhangsun et al., 2007). So we adopted this promoter to make sure that the two target genes would express successfully in transgenic lines. Finally the ELISA results demonstrated that the Ubi1 promoter works well in transgenic lines as anticipated.

ROC22 is an important commercial sugarcane cultivar in China and a lot of genetic improvement project had been done to it. So in this research embryogenic calli was induced from this cultivar and used for transformation. Agro-bacterium infection and resistant calli selection process was conducted in accordance to the protocol induced from Syngenta Biotechnology Inc. PMI/Mannose selection system of transgenic sugarcane was firstly established by this company and works very well (Dong et al., 2014). A total of 4.3 g embryogenic calli was collected and infected, and then 33 resistant plantlets were obtained and sampled for PCR detection. Thirty-two of the plantlets were PCR positive for PMI and EPSPS genes, whereas 30 of the shoots were PCR positive for the Cry1Ab gene (**Figure 4**). A total of 30 transgenic lines were PCR positive for all of the three exogenous genes (**Table 2**). Plantlet number of PCR positive of three exogenous genes are different, this means that in some transgenic lines part of T-DNA fragment may fractured and missed during the integration process. This usually happen during the transformation by insertion a single and long T-DNA fragment containing multiple genes (Cao et al., 2005).

Though 30 shoots were PCR positive for both EPSPS gene and Cry1Ab gene, but ELISA analysis showed that only 23 shoots expressed Cry1Ab and EPSPS protein. This means that EPSPS gene and Cry1Ab gene of the other 7 shoots had been silenced. Exogenous gene silence is normal in the transgenic plants, this had been discovered at least 20 years ago (Stam et al., 1997). And in this research about 23% of sugarcane transgenic lines were silenced for two target genes.

After molecular characterization five single copy and ELISA positive transgenic lines were chose for further research. In voluntary feeding bioassay, the growth and development of larvae that fed on stem tissue from transgenic lines were significantly retarded as compared with those of larvae that fed on stem tissue from non-transgenic lines. At the end all the larvae fed on stem tissue from transgenic lines were survived and successfully pupated. In this testing, if more than 4 g of stem tissue from transgenic lines was added to larva fodder medium, the result may different. Some larvae may die off and can’t successfully pupate finally.

Glyphosate tolerance ability was tested by the spray different dilution of Roundup (from 0.1 to 0.5%). All wild-type sugarcane plantlets died even at the lowest Roundup concentration of 0.1%, whereas the transgenic plantlets grew healthily even at a Roundup concentration reaching 0.5%. Higher concentration of Roundup had not been tested, because 0.1–0.2% is the realistic concentration that used in field.

In the field trials, the five single copy transgenic lines exhibited excellent cane borer resistance and herbicide tolerance but poor agronomic traits; in particular, the transgenic plants were shorter and more slender than the non-transformed control plants. None of the lines can be selected for commercial use. As the biggest biotechnology company in the world such as Syngenta and Monsanto, they usually product hundreds of transgenic lines for one vector, and at the end one or two were chose and tried for commercial used. Therefore, a considerable number of embryogenic calli should be infected to produce transgenic lines with potential for commercial use.

The present study revealed that it is possible to transform Cry1Ab gene and EPSPS gene together to sugarcane cultivar and obtain transgenic sugarcane lines that exhibit excellent cane borer resistance and herbicide tolerance. But considerable number of embryogenic calli should be infected to produce transgenic lines with potential for commercial use.

## Author Contributions

WW took charge of genetic transformation and paper writing. BY and SZ are the directors of the original research and paper writing. XF and CF took charge of insect bioassay and herbicide resistant testing of transgenic lines. JW took charge of vector construction and molecular analysis of transformed plants. ZC and TZ took charge of original paper revising. GX and JZ took charge of field investigation of transgenic plants. LS took charge of data analysis and picture making.

## Conflict of Interest Statement

The authors declare that the research was conducted in the absence of any commercial or financial relationships that could be construed as a potential conflict of interest.
